# Sex-Specific Linear Polyubiquitination Is a Critical Regulator of Contextual Fear Memory Formation

**DOI:** 10.3389/fnbeh.2021.709392

**Published:** 2021-07-09

**Authors:** Madeline Musaus, Kayla Farrell, Shaghayegh Navabpour, W. Keith Ray, Richard F. Helm, Timothy J. Jarome

**Affiliations:** ^1^School of Neuroscience, Virginia Polytechnic Institute and State University, Blacksburg, VA, United States; ^2^Department of Animal and Poultry Science, Virginia Polytechnic Institute and State University, Blacksburg, VA, United States; ^3^Department of Translational Biology, Medicine and Health, Virginia Polytechnic Institute and State University, Roanoke, VA, United States; ^4^Department of Biochemistry, Virginia Polytechnic Institute and State University, Blacksburg, VA, United States

**Keywords:** ubiquitin, memory, amygdala, RNF31, siRNA

## Abstract

Strong evidence supports that protein ubiquitination is a critical regulator of fear memory formation. However, as this work has focused on protein degradation, it is currently unknown whether polyubiquitin modifications that are independent of the proteasome are involved in learning-dependent synaptic plasticity. Here, we present the first evidence that atypical linear (M1) polyubiquitination, the only ubiquitin chain that does not occur at a lysine site and is largely independent of the proteasome, is critically involved in contextual fear memory formation in the amygdala in a sex-specific manner. Using immunoblot and unbiased proteomic analyses, we found that male (49) and female (14) rats both had increased levels of linear polyubiquitinated substrates following fear conditioning, though none of these protein targets overlapped between sexes. In males, target protein functions involved cell junction and axonal guidance signaling, while in females the primary target was Adiponectin A, a critical regulator of neuroinflammation, synaptic plasticity, and memory, suggesting sex-dependent functional roles for linear polyubiquitination during fear memory formation. Consistent with these increases, *in vivo* siRNA-mediated knockdown of *Rnf31*, an essential component of the linear polyubiquitin E3 complex LUBAC, in the amygdala impaired contextual fear memory in both sexes without affecting memory retrieval. Collectively, these results provide the first evidence that proteasome-independent linear polyubiquitination is a critical regulator of fear memory formation, expanding the potential roles of ubiquitin-signaling in learning-dependent synaptic plasticity. Importantly, our data identify a novel sex difference in the functional role of, but not a requirement for, linear polyubiquitination in fear memory formation.

## Introduction

The ubiquitin-proteasome system (UPS) is responsible for controlling the majority of protein degradation in cells (Hershko and Ciechanover, 1998; Jarome and Devulapalli, 2018). In this system, target proteins are “tagged” by the small protein modifier ubiquitin through the coordinated actions of ubiquitin ligases, including the ubiquitin-activating enzyme (E1), ubiquitin carrier enzymes (E2), and ubiquitin conjugation ligases (E3). Most proteins receive several ubiquitin molecules that link together at specific sites on the previous ubiquitin, forming polyubiquitin chains. The lysine-48 (K48) polyubiquitin chain is the canonical protein degradation signal and is the most well-studied and abundant polyubiquitin linkage site in cells (Akutsu et al., 2016). Additionally, numerous studies have shown that dysregulation of the UPS is associated with a wide variety of neurological and neurodegenerative disorders (Dennissen et al., 2012; Zheng et al., 2016), many of which are associated with memory loss, emphasizing its importance in basic and disease-related cellular functioning.

Recent evidence supports that the UPS-mediated protein degradation is a critical regulator of synaptic plasticity and memory formation (Jarome and Helmstetter, 2013; Hegde, 2017; Park and Kaang, 2019). Learning-dependent increases in degradation-specific K48 polyubiquitination have been reported for a variety of behavioral tasks, and administration of catalytic inhibitors of the proteasome impair long-term memory (Lopez-Salon et al., 2001; Artinian et al., 2008; Lee et al., 2008; Jarome et al., 2011; Rodriguez-Ortiz et al., 2011; Reis et al., 2013; Figueiredo et al., 2015; Furini et al., 2015; Werner et al., 2015; Rosenberg et al., 2016b; Cullen et al., 2017; Orsi et al., 2019). Despite this rapidly emerging evidence, the involvement of other polyubiquitin linkage sites, particularly those independent of the proteasome, in memory formation has yet to be explored.

Outside of protein degradation, the UPS performs a variety of other cellular functions, primarily through a number of different types of polyubiquitin chains (reviewed in, Musaus et al., 2020). For example, while lysine 11 (K11) and 48 (K48) polyubiquitin chains are primarily involved in protein degradation, lysine 63 (K63) polyubiquitin chains have been shown to be involved in a variety of cellular processes that include the DNA damage response, intracellular trafficking, and endocytosis (Nathan et al., 2013; Erpapazoglou et al., 2014; Lee et al., 2017). The linear (M1) polyubiquitin chain has recently been shown to be involved in transcriptional regulation *via* a protein-degradation independent mechanism (Rieser et al., 2013; Spit et al., 2019). This linear polyubiquitin chain is unique as it is the only ubiquitin linkage site that does not occur at a lysine, instead forming through the first methionine on the N-terminal. Furthermore, unlike other linkage sites, this atypical linear polyubiquitin chain is formed by only a single E3 ligase, known as the LUBAC complex, which consists of the essential components RNF31 and SHARPIN (Tokunaga et al., 2011; Stieglitz et al., 2013). However, while this polyubiquitin chain has been examined in the immune response (Haas et al., 2009; Gerlach et al., 2011; Emmerich et al., 2013; Iwai et al., 2014; Tang et al., 2018), only a single study has examined the presence of liner polyubiquitination in the brain (Orsi et al., 2019). Importantly, to date, no study has examined the potential role of linear polyubiquitination in the brain during activity and learning-dependent synaptic plasticity.

In the present study, we tested our hypothesis that linear polyubiquitination is a critical regulator of fear memory formation in the amygdala of males and females. We tested this using a combination of rodent behavioral procedures with unbiased proteomic analyses and genetic manipulations and present the first evidence that linear polyubiquitination contributes to fear memory formation in the amygdala in a sex-specific manner. Our results present the first evidence of a role for proteasome-independent protein polyubiquitination in learning-dependent synaptic plasticity and suggest that sex-specific linear polyubiquitination is a critical regulator of fear memory formation in the amygdala.

## Materials and Methods

### Subjects

All experiments used 8–9 week old male or female Sprague–Dawley rats weighing 225–249 or 175–199 g at the time of arrival, respectively, obtained from Envigo (Frederick, MA, USA). Animals were housed two per cage with free access to water and rat chow. The colony was maintained under a 12:12-h light/dark cycle. All experiments took place during the light portion of the cycle. All animal procedures were approved by the Virginia Polytechnic Institute and State University Institutional Animal Care and Use Committee (protocol #18-019) and conducted with the ethical guidelines of the National Institutes of Health.

### siRNA Preparation

Fresh Accell (Horizon, Lafayette, CO, USA) SMARTpool Rnf31 (#E-087475-00-0005) and Sharpin (#E-097217-00-0005) or nontargeting control (#D-001910-10-05) siRNA stocks (100 μM) were resuspended in Accell siRNA delivery media (#B-005000-100) to a concentration of ~9 μM on the day of surgery. A nontargeting green fluorescent Accell siRNA (#D-001950-01-05) was used to confirm the targeting of the BLA.

### Surgery

Rats underwent stereotaxic surgeries where Accell siRNAs were injected into the basolateral amygdala (BLA) using coordinates relative to Bregma (A/P: −3.0, M/L: ±5.0, D/V: −7.7). Animals were anesthetized with 1.5–4% isoflurane and received bilateral injections into the amygdala using a 26-gauge Hamilton syringe connected to an automated pump (Harvard Apparatus, Cambridge, MA, USA) at a rate of 0.1 μl per minute for a total of 0.5 μl per hemisphere. Animals received a subcutaneous injection of carprofen (5 mg/kg) and topical lidocaine on the day of surgery.

### Apparatus

The two identical Habitest chambers used for contextual fear conditioning have been previously described in detail (Orsi et al., 2019). The Habitest chamber consisted of a steel test cage with front and back Plexiglas walls and a grid shock floor above a plastic drop pan. The right wall of the chamber consisted of a house light in the top back corner, which remained on during the behavioral procedures, and an infrared light in the top middle, which was not illuminated during this project. The left wall of the chamber consisted of a high-bright light, which was not illuminated during this project. All remaining slots of both walls were filled with blank metal panels. A USB camera was mounted on a steel panel outside the back Plexiglas wall of the chamber, angled at ~45 degrees. The entire chamber was housed in an isolation cubicle with an acoustic liner and a house fan, which remained active during all behavioral procedures. The shock was delivered through the grid floor *via* a Precision Animal Shocker under the control of FreezeFrame 4 software, which also analyzed animal behavior in real-time. A freezing threshold of 2.0 was used as the scoring parameter for all animals, which we found matches hand-scoring procedures. All video was recorded and stored for later analysis. The chamber walls were wiped with 70% isopropanol before use.

### Behavioral Procedures

Rats underwent contextual fear conditioning training and testing as described previously (Devulapalli et al., 2019; Orsi et al., 2019) in a Habitest chamber. Animals were handled for 4 days prior to behavioral training; the first 2 days occurred in the animal housing room and the second 2 days occurred in an adjacent room where behavioral training was to occur. Following this, animals were placed into the fear conditioning apparatus and after a 1 min baseline, received four unsignaled footshock (1.0 mA, 1 s 59 s ITI) presentations. After a 1 min post-shock period, the animals were returned to their homecages. Importantly, we recently reported that male and female Sprague–Dawley rats perform similar on this task and do not differ in shock reactivity (Devulapalli et al., 2021), eliminating concerns of any biochemical effects seen between sexes being due to differences in behavioral performance or sensory processing. For testing, which occurred 24 h after training, animals were placed back into the training context for 5 min in the absence of shock, unless otherwise noted below. Male and female animals underwent identical procedures.

### Specific Experimental Procedures

#### Experiment 1

Male (*n* = 10) rats were trained to contextual fear conditioning and brain tissue collected 1 h later. Separate male rats (*n* = 9) that were not exposed to the training context and had brain tissue collected from them during the training day served as naïve controls. Collected brain regions were used for protein assays and were compared for linear polyubiquitination in nuclear and cytoplasmic fractions.

#### Experiment 2

Female (*n* = 10) rats were trained to contextual fear conditioning and brain tissue collected 1 h later. Separate female rats (*n* = 10) that were not exposed to the training context and had brain tissue collected from them during the training day served as naïve controls. Collected brain regions were used for protein assays and were compared for linear polyubiquitination in nuclear and cytoplasmic fractions.

#### Experiment 3

Female (*n* = 5) rats were trained to contextual fear conditioning and brain tissue collected 1 h later. Separate female rats (*n* = 5) that were not exposed to the training context and had brain tissue collected from them during the training day served as naïve controls. Collected brain regions were used for TUBE-based mass spectrometry analysis.

#### Experiment 4

Male (*n* = 5) rats were trained to contextual fear conditioning and brain tissue collected 1 h later. Separate male rats (*n* = 5) that were not exposed to the training context and had brain tissue collected from them during the training day served as naïve controls. Collected brain regions were used for TUBE-based mass spectrometry analysis.

#### Experiment 5

Male rats were stereotaxically injected with Accell siRNA targeting *Rnf31* and *Sharpin* (*n* = 7) or control (*n* = 8) into the basolateral amygdala. Five days later, animals were trained to contextual fear conditioning and the following day re-exposed to the training context to assess memory retention. Twenty-four hours after testing, basolateral amygdala tissue was collected, hemispheres were split and processed for protein and RNA assays. Protein assays consisted of proteasome activity and linear polyubiquitination. RNA assays consisted of qRT-PCR for *Rnf31* and *Sharpin*.

#### Experiment 6

Female rats were stereotaxically injected with Accell siRNA targeting *Rnf31* (*n* = 8) or control (*n* = 9) into the basolateral amygdala. Five days later, animals were trained to contextual fear conditioning and the following day re-exposed to the training context to assess memory retention.

#### Experiment 7

Male rats were trained to contextual fear conditioning and 24 h later stereotaxically injected with Accell siRNA targeting *Rnf31* (*n* = 5) or control (*n* = 5) into the basolateral amygdala. Five days later, animals were re-exposed to the training context for 3 min to assess memory retention.

### Tissue Collection

Rats were overdosed on isoflurane in a necrosis chamber and the brain was rapidly removed and immediately frozen on dry ice. Tissue containing the basolateral amygdala (BLA) was then dissected out by blocking the brain in a rat brain matrix (Harvard Apparatus, Holliston, MA, USA) incubated with dry ice. Amygdala hemispheres were split so that one was used for protein extractions and the other for RNA/DNA extractions, which were counterbalanced across extraction procedures to account for any possible laterality effects. All dissected tissue was frozen at −80°C until needed.

### Nuclear and Cytoplasmic Extractions

Nuclear and cytoplasmic extracts from the BLA were collected using our previously described protocol (Devulapalli et al., 2019; McFadden et al., 2019; Orsi et al., 2019). The BLA tissue was homogenized with Teflon tissue grinders using 15 strokes in 500 μl of lysis buffer (10 mM HEPES, 1.5 mM MgCl_2_, 10 mM KCl, 0.5 mM DTT, 0.5% IGEPAL, 1 μl/ml protease inhibitor cocktail, and 1 μl/ml phosphatase inhibitor cocktail) and incubated on ice for 30 min. The homogenized samples were then centrifuged for 10 min at 845× *g* at 4°C. The supernatant was collected as the cytoplasmic fractions. Pelleted nuclei were resuspended in 75 μl of extraction buffer (20 mM HEPES, 6.25% glycerol, 1.5 mM MgCl_2_, 300 mM NaCl, 0.25 mM EDTA, 0.5 mM DTT, 1 μl/ml protease inhibitor cocktail, and 1 μl/ml phosphatase inhibitor cocktail) and mixed by pipetting. The resuspended nuclei were incubated on ice for 30 min and then centrifuged for 20 min at 21,130× *g* at 4°C. The resulting supernatant containing nuclear proteins was collected and protein concentration was determined by using the Bio-Rad (Hercules, CA, USA) Dc protein assay.

### Whole Cell Tissue Collection

For whole cell tissue collection, samples were lysed in buffer (10 mM HEPES, 1.5 mM MgCl_2_, 10 mM KCl, 0.5 mM DTT, 0.5% IGEPAL, 0.2% SDS, 1 μl/ml protease inhibitor cocktail, and 1 μl/ml phosphatase inhibitor cocktail), centrifuged for 10 min at 4,000× *g* at 4°C, the supernatant collected and protein concentration was determined by using the Bio-Rad DC protein assay.

### Proteasome Activity Assay

Proteasome activity assays were performed as described previously (Devulapalli et al., 2019; McFadden et al., 2019; Orsi et al., 2019) Normalized samples (10 μg) were diluted in MilliQ H_2_O and mixed with reaction buffer (250 mM HEPES, pH 7.5, 5 mM EDTA, 0.5% NP-40, 0.01% SDS, 5 mM ATP). Fluorogenic peptide Suc-leu-leu-val-thy-AMC (#BML-P802-0005, Enzo Life Sciences) was then added to the samples at a concentration of 10 μM to assess proteasome chymotrypsin-like activity. The reaction was incubated at 37°C for 2 h and fluorescence monitored at 360 (excitation)/460 (emission) on a monochromatic plate reader (Synergy H1; Biotek, Winooski, VT, USA). Protein-free blanks were used and an AMC standard curve was produced. The scan with the peak level of AMC was used for statistical analyses. Data are presented as the percentage change in relative fluorescent units (RFU) relative to the Control group.

### Antibodies

Mouse monoclonal antibodies included linear polyubiquitin (1:2,500; #AB130, Life Sensors, Malvern, PA, USA).

### Western Blots

Western blots were performed with 7% Acrylamide gels using 5–10 μg of protein and transferred with a Turbo Transfer System (Biorad) as described previously (Orsi et al., 2019). Membranes were incubated in a 50:50 blocking buffer (50% Licor TBS blocking buffer and 50% TBS + 0.1% Tween-20) for 1 h at room temperature, followed by overnight incubation in primary antibody in 50:50 blocking buffer at 4°C. Membranes were then washed three times for 10 min with TBS + 0.1% Tween-20 (TBSt) and incubated in secondary antibody (1:20,000; goat anti-mouse IgG2 800CW) in 50:50 blocking buffer for 45 min. After two 10 min washes in TBSt, the membranes were washed in TBS, imaged using the Odyssey Fc (LI-COR, Lincoln, NE, USA) and visualized proteins were analyzed using Image Studio Ver 5.2. Samples were normalized to Coomassie blue, which consisted of staining membranes for 10 s washing extensively in 50% methanol, and imaging at 700CW using the Odyssey Fc. All optical densities were taken from the entire length of the molecular standards ladder for the linear polyubiquitin developments.

### Tandem Ubiquitin Binding Entity (TUBE) Assay

BLA tissue was homogenized in buffer (10 mM HEPES, 1.5 mM MgCl_2_, 10 mM KCl, 0.5 mM DTT, 0.5% IGEPAL, 0.02% SDS, 70 mM NEM, 1 μl/ml protease inhibitor cocktail, and 1 μl/ml phosphatase inhibitor cocktail). The concentration of NEM used has been shown to preferentially preserve linear polyubiquitinated proteins during tissue lysis (Emmerich and Cohen, 2015). Then a linear polyubiquitin-selective tandem ubiquitin binding entity (TUBE) assay was performed. MagnaLink streptavidin magnetic beads (#M-1003-010; Vector Laboratories, Burlingame, CA, USA) were aliquoted (100 μl), washed thoroughly with Wash Buffer (100 mM Tris-HCL, 150 mM NaCl, 5 mM EDTA, 0.08% NP-40) and 4 μl of M1-specific TUBE (#UM-0306-0050; Life Sensors, Malvern, PA) was added, followed by incubation for 2 h on rotator at 4°C. Beads were then washed and a 500 μl mixture of protein (300 μg for all samples and both sexes), protease inhibitor (10 μg/μl), and Wash Buffer was added to each tube, followed by incubation for 2 h on rotator at 4°C. Samples were then washed twice and incubated at 96°C for 5 min at 800 rmp in 1X sample buffer. After cooling at room temperature, the supernatant was collected and stored at −80°C for mass spectrometry analysis.

### Liquid Chromatography Mass Spectrometry (LC/MS)

Optima^TM^ LC/MS grade solvents, Pierce^TM^ trypsin protease (MS grade), were from Fisher Scientific (Waltham, MA, USA). S-Trap^TM^ micro columns were from ProtiFi (Farmingdale, NY, USA). Triethylammonium bicarbonate, pH 8.5 (TEAB), *o*-phosphoric acid, and formic acid were from MilliporeSigma (St. Louis, MO, USA).

Protein samples were acidified by the addition of 11.1 μl 12% (v/v) *o*-phosphoric acid then protein precipitated by the addition of 725 μl LC/MS grade methanol and incubation at −80°C overnight. Precipitated protein was collected at the bottom of the sample tubes by centrifugation at room temperature for 15 min at 13,000× *g*. All but approximately 150 μl of the liquid from each sample was removed and discarded. Precipitated protein in each sample tube was then homogenized in the remaining liquid by scraping the sides of the tube with a pipette tip and repeated pipetting. The protein homogenate from each sample was then loaded onto an S-Trap^TM^ micro column by centrifugation at room temperature for 1 min at 1,000× *g*. Each S-Trap^TM^ micro column was washed four times with 150 μl LC/MS grade methanol at room temperature for 1 min at 1,000× *g*. Pierce^TM^ trypsin protease (0.8 μg in 25 μl 50 mM TEAB) was loaded on top of each S-Trap^TM^ micro column and incubated for 4 h at 37°C. A second aliquot of trypsin (0.8 μg in 25 μl 50 mM TEAB) was loaded on top of each S-Trap^TM^ micro column and incubated overnight at 37°C.

Peptides were recovered by sequential washing of the spin column with 25 μl 50 mM TEAB, 25 μl solvent A [2:98 LC/MS grade acetonitrile: LC/MS grade water supplemented with 0.1% (v/v) formic acid], and 25 μl solvent B [80:20 LC-MS grade acetonitrile: LC-MS grade water supplemented with 0.1% (v/v) formic acid]. Acetonitrile was removed using a centrifugal vacuum concentrator, then peptide concentration was determined by measuring the absorbance at 215 nm using a DS-11 FX+ spectrophotometer/fluorometer (DeNovix, Wilmington, DE, USA). Samples were diluted to 0.5 mg/ml using solvent A and 2 μl (1 μg, females) or 4 μl (2 μg, males) were analyzed using LC-MS/MS and each sample was analyzed twice yielding technical duplicates. The higher concentration used for males was due to a greater streptavidin signal than seen in females. As noted below, final values are normalized to streptavidin to account for this difference.

Samples were first loaded onto a precolumn [Acclaim PepMap 100 (Thermo Scientific, Waltham, MA, USA), 100 μm × 2 cm] after which flow was diverted to an analytical column [50 cm μPAC (PharmaFluidics, Woburn, MA, USA)]. The UPLC/autosampler utilized was an Easy-nLC 1200 (Thermo Scientific, Waltham, MA, USA). The flow rate was maintained at 150 nl/min and peptides were eluted utilizing a 2–45% gradient of solvent B in solvent A over 88 min. The mass spectrometer utilized was an Orbitrap Fusion Lumos Tribid^TM^ from Thermo Scientific (Waltham, MA, USA). Spray voltage on the μPAC compatible Easy-Spray emitter (PharmaFluidics, Woburn, MA, USA) was 1,300 volts, the ion transfer tube was maintained at 275°C, the RF lens was set to 30%, and the default charge state was set to 3.

MS data for the m/z range of 400–1,500 was collected using the orbitrap at 120,000 resolution in positive profile mode with an AGC target of 4.0e5 and a maximum injection time of 50 ms. Peaks were filtered for MS/MS analysis based on having isotopic peak distribution expected of a peptide with an intensity above 2.0e4 and a charge state of 2–5. Peaks were excluded dynamically for 15 s after 1 scan with the MS/MS set to be collected at 45% of a chromatographic peak width with an expected peak width (FWHM) of 15 s MS/MS data starting at m/z of 150 was collected using the orbitrap at 15,000 resolution in positive centroid mode with an AGC target of 1.0e5 and a maximum injection time of 200 ms. Activation type was HCD stepped from 27 to 33.

Data were analyzed utilizing Proteome Discoverer 2.5 (Thermo Scientific, Waltham, MA, USA) combining a Sequest HT and Mascot 2.7 (Matrix Science, Boston, MA, USA) search into one result summary for each sample. Both searches utilized the UniProt reference *R. norvegicus* proteome database (downloaded July 28, 2020) and a common protein contaminant database provided with the Proteome Discoverer (PD) software package. Each search assumed trypsin-specific peptides with the possibility of two missed cleavages, a precursor mass tolerance of 10 ppm and a fragment mass tolerance of 0.1 Da. Sequest HT searches also included the PD software precursor detector node to identify MSMS spectra containing peaks from more than one precursor. Sequest HT searches included a fixed modification of carbamidomethyl at Cys and the variable modifications of oxidation at Met and loss of Met at the N-terminus of a protein (required for using the INFERYS rescoring node). Peptide matches identified by Sequest HT were subjected to INFERYS rescoring to further optimize the number of peptides identified with high confidence.

Mascot searches included the following dynamic modifications: oxidation of Met, acetylation of the protein N-terminus, cyclization of a peptide N-terminal Gln to pyro-Glu, N-ethylmaleimide at Cys, DeStreak (β-mercaptoethanol) at Cys, GlyGly at Lys, and deamidation of Asn/Gln residues.

Protein identifications were reported at a 1% false discovery rate (high confidence) or at a 5% false discovery rate (medium confidence) based on searches of decoy databases utilizing the same parameters as above. The software matched peptide peaks across all runs and protein quantities are the sum of all peptide intensities associated with the protein. Values were normalized to Streptavidin. Technical duplicates were averaged then biological replicates were averaged before determination of the trained to naïve ratio. A simple *t*-test was used to determine *p*-values comparing the five trained males to the five naïve males and the five trained females to the five naïve females. All data and related files were submitted to the PRIDE archive with accession number PXD025483.

### Mass Spectrometry Pathway Analysis

All quantified ubiquitinated proteins were analyzed for identifying pathways and networks using Ingenuity Pathway Analysis (IPA) software (QIAGEN, Redwood City, CA, USA) core analysis based on the user dataset as the reference set. The top canonical pathways and molecular networks associated with the uploaded dataset were listed along with the *p*-values calculated using a right-tailed Fisher’s exact test. Activation z-scores were calculated by IPA’s z-score algorithm to predict the overall inhibition or activation of the identified pathways/networks. A positive z-score implies an overall predicted activation of the pathway/molecular networks (or an increase in the ubiquitination levels of the proteins associated with them), while a negative z-score implies an overall predicted inhibition or downregulation of the pathway/molecular networks. Cellular processes/pathways with no z-scores imply that IPA was unable to generate prediction states for these functionalities.

### RNA/DNA Extractions

RNA was extracted from BLA lysates using the Qiagen (Germantown, MD, USA) Allprep kit, according to the manufacturer’s instructions. RNA concentration was measured on the Take3 (BioTek, Winooski, VT, USA), normalized (200 ng), and converted to cDNA using the iScript cDNA synthesis kit (Bio-rad).

### Quantitative Real-Time PCR

Real-time PCR amplifications of the cDNA were performed on the Bio-rad CFX96 Real-Time System using the following protocol: 95.0°C for 3 min, then 95.0°C for 10 s, followed by 60°C for 30 s (39 repeats), 55–95°C for 0.5°C/cycle, followed by a melt curve starting at 55.0°C for 10 s (81 repeats), and then held at 4.0°C. Primers were *Rnf31* (F: GGGTCCTCACAACACCTCAG; R: TGTCTATCCAGGCCATCCCA), and *Sharpin* (F: GGTCCCATAAGGCTGCAAGT; R: GCAGTAAGGGGATCCCAAGC). *Gapdh* (F: ACCTTTGATGCTGGGGCTGGC; R: GGGCTGAGTTGGGATGGGGACT) was used as an internal control and data were analyzed using the comparative Ct method.

### Statistical Analyses

All data are presented as mean with standard error, with scatter plots to identify individual samples (except in line graphs). Data were analyzed with the two-tailed *t*-test, except for training data which were analyzed with 2-way ANOVA and Fisher LSD posthoc tests. Statistical outliers were defined as those samples that were two or more standard deviations from the mean and were determined by the outlier function in Prism.

## Results

### Linear Polyubiquitination Is Globally Increased in the Amygdala of Males, but Not Females, After Contextual Fear Conditioning

**Figure 1:**
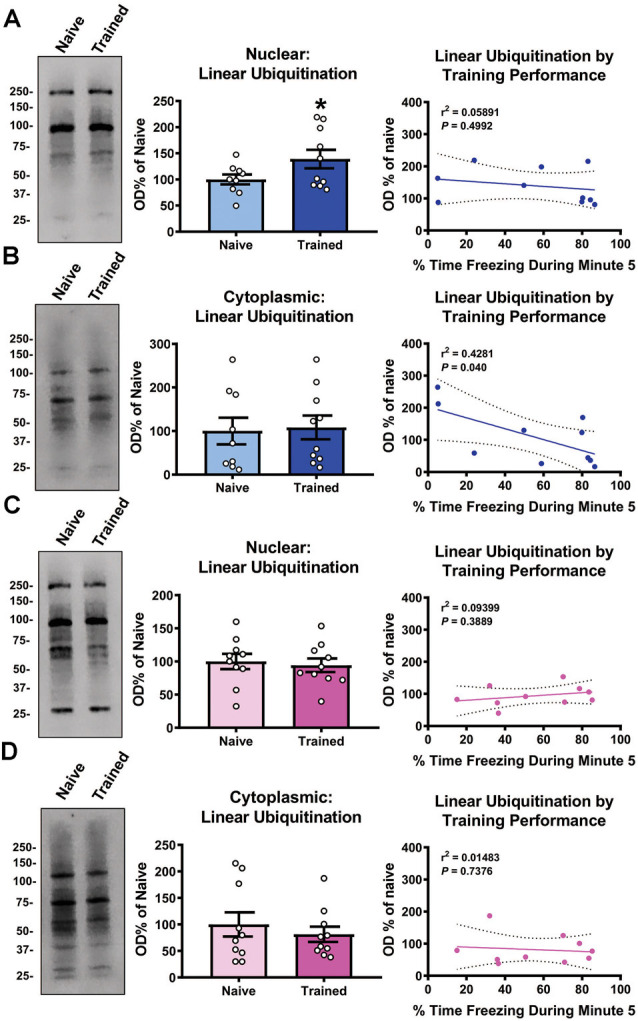
Global levels of nuclear linear polyubiquitination are increased in the amygdala of male, but not female, rats following fear conditioning. Male and female rats were trained on a contextual fear conditioning paradigm and nuclear and cytoplasmic extracts from the basolateral amygdala (BLA) were collected 1 h later for protein analysis. These samples were compared with BLA tissue collected from naïve rats. **(A,B)** In males, linear polyubiquitination was increased in the nucleus **(A)**, but not cytoplasm **(B)**, of BLA cells following fear conditioning. **(C,D)** In females, levels of linear polyubiquitination were not altered in the nucleus **(C)** or cytoplasm **(D)** of BLA cells following fear conditioning. Correlational analyses between linear polyubiquitination levels in the Trained group and the final minute of the training session are shown to the right of each bar graph. All western blots were normalized to Coomassie blue stain on the membrane. *N* = 9–10 per group per sex. *P < 0.05 from Naïve.

We recently reported that linear polyubiquitination increased in the nucleus of cells within the basolateral amygdala (BLA) in a learning-specific manner 1 h after contextual fear conditioning (Orsi et al., 2019). To confirm this, we trained male rats to a contextual fear conditioning procedure and collected BLA nuclear and cytoplasmic fractions 1 h later and immunoblotted with an M1-specific polyubiquitin antibody (Matsumoto et al., 2012). Associative control animals were not used as we have previously shown that these conditions do not engage linear polyubiquitination in the amygdala (Orsi et al., 2019). We found a main effect for Time (*F*_(4,68)_ = 39.95, *P* < 0.0001), but not Sex (*F*_(1,17)_ = 0.4586, *P* = 0.5074), and there was not a Time × Sex interaction (*F*_(4,68)_ = 0.1596, *P* = 0.9580), indicating that male and female rats did not differ in behavioral performance during the training session (Table 1). Consistent with our previous work, we found that nuclear (*t*_17_ = 1.883, *P* = 0.0385; Figure 1A), but not cytoplasmic (*t*_17_ = 0.1981, *P* = 0.8453; Figure 1B), linear polyubiquitination increased in the BLA following fear conditioning. These results support that nuclear linear polyubiquitination levels increase as a function of fear learning in the amygdala. As our previous work only used male rats, we repeated the experiment using female rats. Surprisingly, we found that nuclear (*t*_18_ = 0.3738, *P* = 0.7129; Figure 1C) and cytoplasmic (*t*_18_ = 0.6873, *P* = 0.5006; Figure 1D) linear polyubiquitination levels did not change in the BLA of female rats following fear conditioning, which is likely due to the high resting levels of ubiquitination typically seen in the amygdala of female rats (Devulapalli et al., 2021). In order to further examine the relationship between linear polyubiquitination levels and behavioral performance, we next performed correlational analyses. We observed a significant correlation in linear polyubiquitination with performance during the final minute of the training session in the male cytoplasmic fraction (*r*^2^ = 0.4281, *P* = 0.040; Figure 1B), which was not found in the male nuclear (*r*^2^ = 0.05891, *P* = 0.4992; Figure 1A), female nuclear (*r*^2^ = 0.09399, *P* = 0.3889; Figure 1C), and cytoplasmic fractions (*r*^2^ = 0.01483, *P* = 0.7376; Figure 1D). Collectively, these results indicate that males, but not females, broadly engage linear polyubiquitination in the amygdala following fear conditioning.

**Table 1:** Performance (% time freezing) during training (Mean ± SEM).

Sex	Minute 1	Minute 2	Minute 3	Minute 4	Minute 5
Male	3.49 ± 0.44	13.05 ± 4.07	34.33 ± 7.41	53.18 ± 9.03	61.37 ± 9.87
Female	2.61 ± 0.45	12.13 ± 2.94	29.35 ± 6.68	45.55 ± 6.05	55.91 ± 7.89

### Sex-Specific Protein Targets of Linear Polyubiquitination After Fear Conditioning

**Figure 2:**
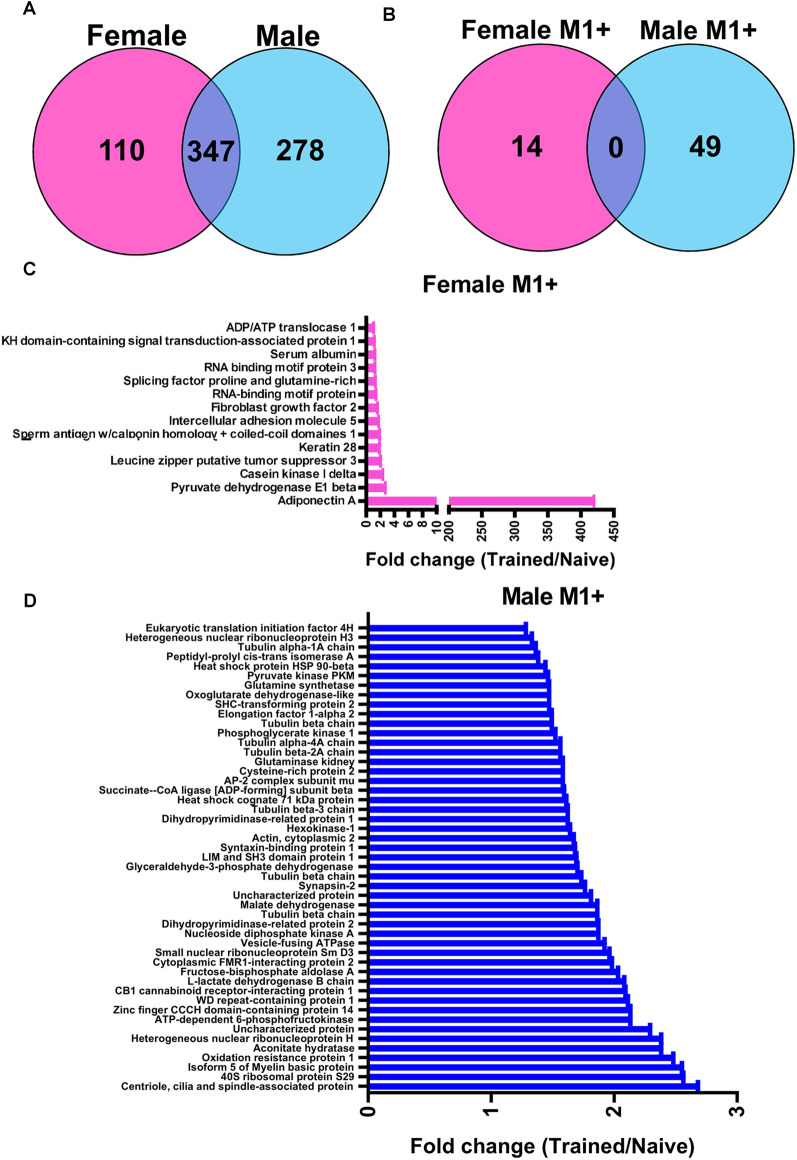
Sex-specific protein targets of linear polyubiquitination in the amygdala following fear conditioning. Male and female rats were trained on a contextual fear conditioning paradigm and protein extracts from the BLA were collected 1 h later. Samples were then purified with a linear polyubiquitin-specific Tandem Ubiquitin Binding Entity (TUBE) and liquid chromatography mass spectrometry performed. These samples were compared with BLA tissue collected from naïve rats. **(A)** Venn diagrams showing the total number of identified linear polyubiquitinated proteins in female (pink) and male (blue) rats. **(B)** Venn diagrams showing the protein targets that gained linear polyubiquitination following fear conditioning in female (pink) and male (blue) rats. **(C,D)** Graph summarizing the protein targets that gained linear polyubiquitin modification following fear conditioning in female **(C)** and male **(D)** rats. Proteins are identified on the *y*-axis and fold change in linear polyubiquitination (Trained/Naïve) is on the *x*-axis. *N* = 5 per group per sex.

We next wanted to identify the potential role of linear polyubiquitination in fear memory formation, which can be inferred by determining its protein targets. Additionally, while females did not show increases in global levels of linear polyubiquitination in the BLA following fear conditioning, it is possible that individual proteins do acquire this ubiquitin modification following learning but are not able to be detected in the western blot assay that only measures global levels of this mark. To address this, we collected BLA lysates from male and female rats 1 h after fear conditioning (separate experiments), purified them with an M1-specific Tandem Ubiquitin Binding Entity (TUBE; Hjerpe et al., 2009), and performed unbiased proteomic analysis using liquid chromatography mass spectrometry (LC/MS). In total, we identified 457 and 625 linear polyubiquitinated proteins in the amygdala of female and males rats, respectively, of which 347 (47.2%) overlapped between sexes (Figure 2A). Of these, we identified 63 unique proteins that have increased linear polyubiquitination following fear conditioning, with 14 targets identified in females and 49 in males (Figure 2B). Conversely, both sexes had only three proteins that lost linear polyubiquitin modifications after training. Importantly, despite the nearly 50% overlap in linear polyubiquitinated protein targets between our datasets, none of the total 69 significant proteins (increased and decreased targeting) identified overlapped between sexes, suggesting that males and females show unique increases and decreases in linear polyubiquitin protein targeting following fear conditioning. In females, the learning-induced increases in linear polyubiquitination primarily occurred on one protein, Adiponectin A, which had a 421-fold increase relative to Naïve controls (Figure 2C). No other protein showed a fold increase over three. Conversely, in males linear polyubiquitination increases occurred in a relatively uniform manner across the 49 identified targets (Figure 2D). These results suggest that while males have broad increases in linear polyubiquitination with fear conditioning, in females these changes are largely centered around a single protein, which likely explains the sex difference observed in our western blot analysis (Figure 1).

**Figure 3:**
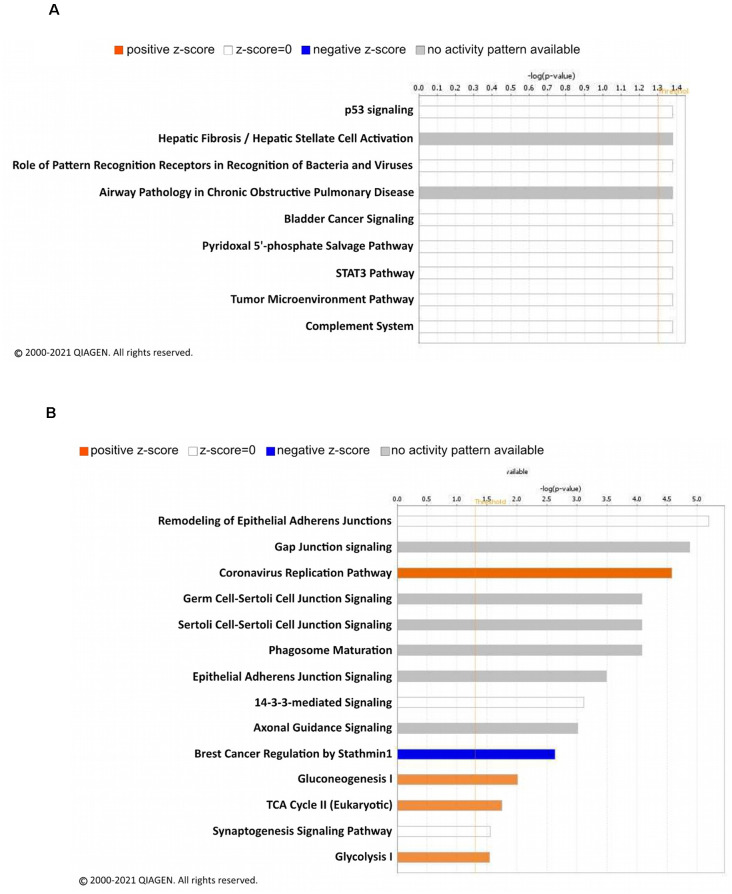
Canonical signaling pathway analysis of linear polyubiquitin protein targets in the amygdala of male and female rats following fear conditioning. Ingenuity pathway analysis (IPA) was performed on samples that underwent mass spectrometry analysis, as described in Figure 2. The top canonical pathways relevant to the regulated linear polyubiquitinated proteins in females **(A)** and males **(B)** are shown with their corresponding score (-log[p value]). A positive z-score indicates the predicted upregulation of specific cellular and molecular processes.

To decipher signaling pathways regulated by the changes of the linear ubiquitination levels due to fear learning, we performed a core analysis of the ubiquitinated proteins using the IPA software. In females, nine different canonical signaling pathways were identified (Figure 3A), including p53 signaling and the STAT3 pathway, though all of these were largely driven by a single protein and by definition did not reach the level needed for a functional network. Four functional networks were identified to be associated with the differentially ubiquitinated proteins in our dataset in males, including behavior, cell-cell signaling and interaction, cell cycle, and nervous system development and function. In addition to these functional networks, 14 canonical signaling pathways were also identified, of which several of the differentially ubiquitinated proteins in our dataset were members of (Figure 3B). Importantly, the majority of these pathways were involved in junction signaling, suggesting a common function of linear polyubiquitination in males during fear memory formation.

**Figure 4:**
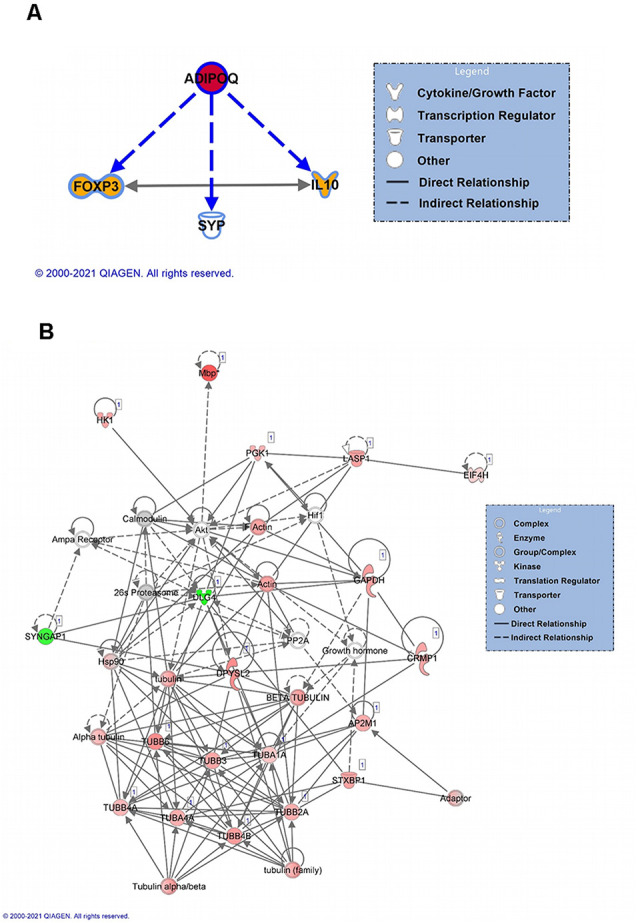
Network analysis of linear polyubiquitin protein targets in the amygdala of male and female rats following fear conditioning. IPA analysis was performed on samples that underwent mass spectrometry analysis, as described in Figure 2. **(A)** IPA analyses of downstream targets of Adiponectin protein with increased ubiquitination in females. The color red indicates an increase in the ubiquitination levels of Adiponectin, and the color orange indicates an activation of the target proteins. **(B)** Functional network of protein-protein interaction by IPA analysis in males. Using IPA, a protein network of proteins differentially ubiquitinated and other associated proteins was created. Solid lines refer to direct and dotted lines represent indirect directions between the proteins. The color red indicates increased ubiquitination, with shading denoting significance level, whereas the proteins with decreased ubiquitination levels are shown in green. Gray denotes proteins that are part of the network but not identified as targets of linear polyubiquitination in our LC/MS analysis. The protein-protein interactions are shown by arrows.

For females, we next focused on the main identified protein in our analysis, Adiponectin A (ADIPOQ), to identify its known downstream targets. Our analysis indicated that ADIPOQ is a positive regulator of forkhead box protein 3 (FOXP3) and Interleukin 10 (IL10) function (Figure 4A), which bidirectionally regulate each other and are involved in the neuroinflammation response. This suggests that in females linear polyubiquitination may be primarily involved in the regulation of ADIPOQ activity and potentially its downstream effects on neuroinflammation in the amygdala following fear conditioning. As males had a larger number of protein targets with a similar degree of linear polyubiquitination following fear conditioning, we instead performed a protein-interaction network analysis (Figure 4B). The color red indicates increased ubiquitination, whereas the proteins with decreased ubiquitination levels are shown in green. Interestingly, we found that more than half of these target proteins could fit into a single network. Importantly, most of these proteins were structural, largely consisting of Tubulin variants, and/or considered to be housekeepers. This network also included the 26S proteasome and its critical regulatory factor calcium/calmodulin-dependent protein kinase 2 (CaMKII), both of which have been previously implicated in the protein degradation process that underlies fear memory formation in the amygdala (Jarome et al., 2011, 2013). Collectively, these results suggest that in males linear polyubiquitination may work in concert with the protein degradation process to regulate fear memory formation in the amygdala.

### Both Males and Females Require Linear Polyubiquitination in the Amygdala for Fear Memory Formation

**Figure 5:**
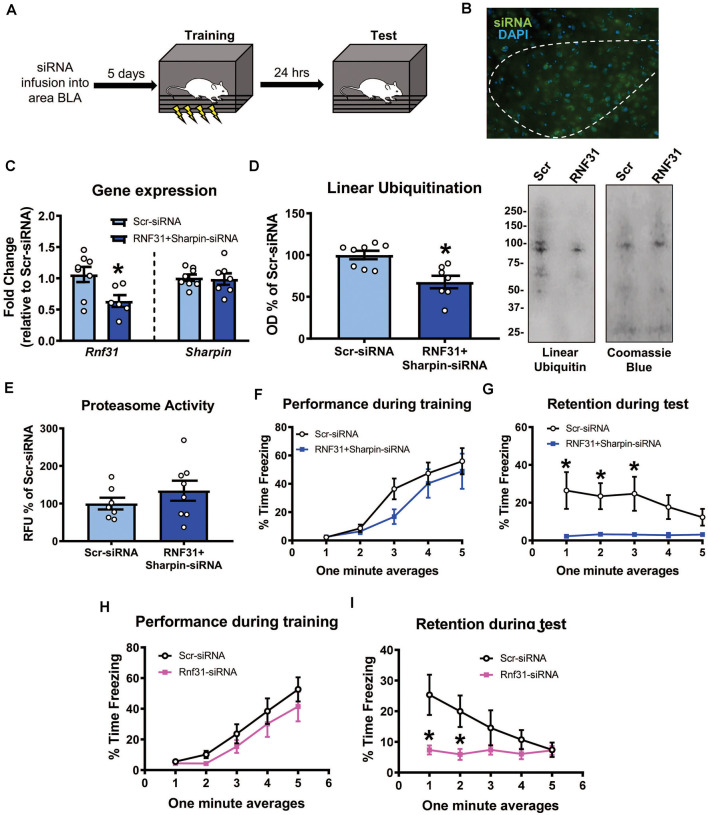
*In vivo* siRNA-mediated knockdown of linear polyubiquitination in the amygdala impairs fear memory in both males and females. **(A)** Male rats received stereotaxic injections of Accell siRNAs targeting *Rnf31* and *Sharpin* 5 days prior to contextual fear conditioning. They were tested for memory to the training context 24 h after training. **(B)** Expression of green Accell siRNA in amygdala 5 days after infusion. **(C–E)** Successful knockdown of *Rnf31*, but not *Sharpin*, expression in the amygdala **(C)**, which reduced baseline linear ubiquitination levels **(D)** without altering proteasome activity **(E)**. **(F,G)** Knockdown of *Rnf31* did not alter performance during the fear conditioning task **(F)** but impaired memory the following day **(G)**. **(H,I)** Knockdown of *Rnf31* in female rats did not alter performance during the fear conditioning task **(H)** but impaired memory the following day **(I)**. All western blots were normalized to Coomassie blue stain. *N* = 7–8 per group for males, 8–9 per group for females. *P < 0.05 from Scr-siRNA.

**Figure 6:**
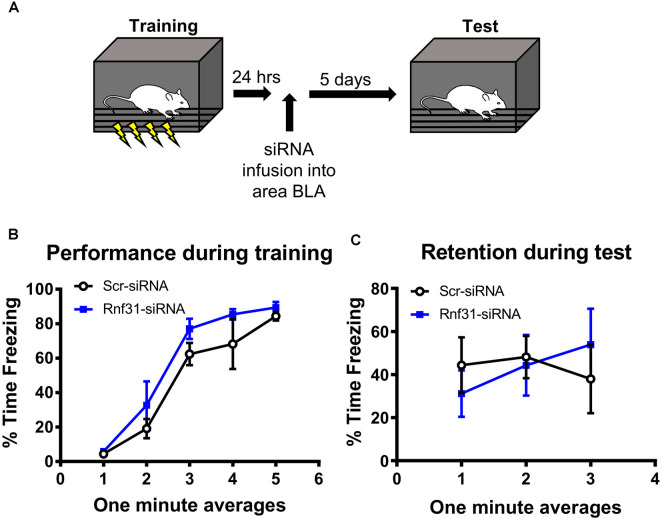
*In vivo* siRNA-mediated knockdown of linear polyubiquitination in the amygdala does not impair fear memory retrieval. **(A)** Male rats were training to contextual fear conditioning and 24 h later received stereotaxic injections of Accell siRNA targeting *Rnf31*. They were tested for memory to the training context 5 days after siRNA infusion. **(B)** Performance during the fear conditioning task. **(C)** Knockdown of *Rnf31* did not alter performance during the test session. *N* = 5 per group.

We next tested whether linear polyubiquitination was critical for fear memory formation in the amygdala. To do this, we stereotaxically injected Accell siRNAs against *Rnf31* and *Sharpin*, two essential components of the E3 ligase LUBAC that conjugates all linear polyubiquitination in cells, into the BLA of male rats. Five days later, we trained animals to contextual fear conditioning and tested memory retention for the task the following day (Figures 5A,B). Our siRNA approach was able to successfully reduce *Rnf31* (*t*_12_ = 2.614, *P* = 0.0226; Figure 5C), but not *Sharpin* (*t*_13_ = 0.2054, *P* = 0.8404; Figure 5C), expression in the BLA. However, consistent with previous studies (Gerlach et al., 2011; Tokunaga et al., 2011; Tang et al., 2018), the loss of *Rnf31* alone was sufficient to decrease linear polyubiquitination levels in BLA whole cell lysates (*t*_13_ = 3.658, *P* = 0.0029; Figure 5D). Importantly, loss of *Rnf31* did not alter proteasome function (*t*_13_ = 1.067, *P* = 0.3053; Figure 5E), suggesting the protein degradation process was unaffected by a loss of linear polyubiquitination. Furthermore, while loss of *Rnf31* had no effect on performance during the fear conditioning task (2 way ANOVA—Time: *F*_(4,52)_ = 24.29, *P* < 0.0001; Group: *F*_(1,13)_ = 1.485, *P* = 0.2447; Interaction: *F*_(4,52)_ = 0.7502, *P* = 0.5624; Figure 5F), it significantly impaired memory the following day (2 way ANOVA—Time: *F*_(4,52)_ = 0.9767, *P* = 0.4283; Group: *F*_(1,13)_ = 8.222, *P* = 0.0132; Interaction: *F*_(4,52)_ = 1.082, *P* = 0.3752; Figure 5G). Next, we confirmed these results in females by manipulating *Rnf31* alone as our *Sharpin* siRNA proved ineffective, which could have caused nonspecific effects in the male animals leading to memory loss. However, consistent with the male data, a loss of *Rnf31* alone in the amygdala of females had no effect on performance during the fear conditioning task (2 way ANOVA—Time: *F*_(4,60)_ = 28.52, *P* < 0.0001; Group: *F*_(1,15)_ = 1.313, *P* = 0.2697; Interaction: *F*_(4,60)_ = 0.3122, *P* = 0.8687; Figure 5H), but significantly impaired memory the following day (2 way ANOVA—Time: *F*_(4,60)_ = 2.748, *P* = 0.0363; Group: *F*_(1,15)_ = 5.673, *P* = 0.0309; Interaction: *F*_(4,60)_ = 2.697, *P* = 0.0391; Figure 5I), suggesting that linear polyubiquitination is a sex-independent regulator of fear memory formation. Finally, to confirm that loss of linear polyubiquitination specifically affected memory consolidation and not retrieval, we delayed siRNA infusions until 24 h after training in male rodents (Figures 6A,B). We found that delaying *Rnf31* knockdown until after the consolidation process was complete did not affect memory retrieval during the test session 5 days later (2 way ANOVA—Time: *F*_(2,16)_ = 0.6890, *P* = 0.5164; Group: *F*_(1,8)_ = 0.0004820, *P* = 0.9830; Interaction: *F*_(2,16)_ = 1.639, *P* = 0.2252; Figure 6C). Collectively, these results suggest that linear polyubiquitination is a critical regulator of fear memory formation in the amygdala of both males and females.

## Discussion

Previous studies have provided convincing evidence that ubiquitin signaling, primarily in the form of protein degradation-specific polyubiquitination, is a critical regulator of memory formation (Lopez-Salon et al., 2001; Artinian et al., 2008; Jarome et al., 2011; Rodriguez-Ortiz et al., 2011; Reis et al., 2013; Felsenberg et al., 2014; Figueiredo et al., 2015; Werner et al., 2015; Rosenberg et al., 2016a, b; Cullen et al., 2017). Furthermore, protein monoubiquitination has also been implicated in the memory formation process (Pavlopoulos et al., 2011). However, to date, no study had examined the role of proteasome-independent protein polyubiquitination in memory formation. Here, we present the first evidence that atypical linear polyubiquitination, which is the only ubiquitin linkage site not occurring at a lysine and is largely independent of the proteasome complex, is a critical regulator of fear memory formation in the amygdala. Importantly, we show that males and females differ in the protein targets and likely functional role of linear polyubiquitination during fear memory formation. Collectively, these results expand our understanding of the role of protein polyubiquitination in memory formation and challenge conventional models that focus largely on protein degradation-specific roles of the UPS in learning-dependent synaptic plasticity. Furthermore, these results expand on the recently reported sex differences in the role of ubiquitin-proteasome signaling in fear memory formation in the amygdala (Devulapalli et al., 2021).

We previously reported that lysine 48 (K48), lysine 63 (K63), and linear polyubiquitination levels increased in the nucleus of amygdala cells of male rats after fear conditioning (Orsi et al., 2019). However, to date, no study had directly manipulated the levels of any specific polyubiquitin chains following learning. Furthermore, all of the evidence implicating the K48 linkage site in memory formation has come *via* studies using pharmacological inhibition of the proteasome, which can indirectly affect all ubiquitin modifications by depleting the free ubiquitin pool. Here, we provide the first direct evidence that linear polyubiquitination is critical for memory formation, which was achieved by manipulation of the only E3 ligase for this linkage site, LUBAC. While it is unclear if other polyubiquitin chains are also critical for memory formation, our data provide a new direction for future studies to examine the role of specific polyubiquitin linkage sites in memory formation, as opposed to broad manipulations of overall ubiquitin-proteasome signaling.

Despite strong evidence that ubiquitin-proteasome signaling is a critical regulator of memory formation, the protein targets of ubiquitin following learning remain largely unknown. To date, the synaptic scaffolds SHANK and GKAP, the RNA-induced silencing complex (RISC) factor MOV10, and the transcriptional repressor IκB have been the only identified targets of proteasome-dependent protein polyubiquitination following learning (Lopez-Salon et al., 2001; Lee et al., 2008; Jarome et al., 2011). Here, we have begun to address this gap by providing the first proteomic analysis of linear polyubiquitin modifications in the brain during fear memory formation. In females, we identified the primary target as ADIPOQ, which in the brain is a known regulator of the neuron inflammation response (Bloemer et al., 2018), likely through control of FOXP3 and IL10 function. Importantly, FOXP3 is ubiquitously expressed in the brain with no observed sex differences (Taylor et al., 2020) and is a known transcription factor and repressor of the well-described memory-permissive NF-κB signaling pathway (Kim, 2009). IL10 is an anti-inflammatory cytokine that is one of the most important mechanisms involved in limiting neuroinflammation (Lobo-Silva et al., 2016). Interestingly, some evidence does support that there is increased neuroinflammation in select brain regions during fear memory formation (Chaaya et al., 2019; Enomoto and Kato, 2021), suggesting that linear polyubiquitination could be involved in regulating this process *via* targeting of ADIPOQ. Furthermore, recent evidence has shown that ADIPOQ regulates long-term potentiation (LTP), long-term depression (LTD) and paired-pulse facilitation in hippocampal slices (Weisz et al., 2017; Pousti et al., 2018), suggesting it plays a critical role in the regulation of synaptic plasticity. Consistent with this, strong evidence suggests that ADIPOQ is critical for memory formation (Ng et al., 2016; Kim et al., 2017). Together, these results suggest that *via* targeting of ADIPOQ, linear polyubiquitination could be involved in controlling neuroinflammation and promoting synaptic plasticity in the amygdala following fear conditioning. However, as ADIPOQ has only recently begun to be investigated in the central nervous system and how it regulates memory formation is unknown, it is also possible that linear polyubiquitination of AIDPOQ could regulate fear memory formation *via* a process that is independent of neuroinflammation. Conversely, in males, this same linear polyubiquitin modification was primarily targeting proteins involved in cell-to-cell communication and may do so by coordinating with the proteasome-mediated protein degradation process. However, it is currently unknown how linear polyubiquitination affected its target proteins and whether it controls activity, cellular localization, protein-protein interactions, or stability. Future studies should aim to better identify how linear polyubiquitination regulates the identified target proteins, which would provide a more detailed understanding of the sex-dependent functional significance of this atypical ubiquitin modification during fear memory formation.

An important outcome of our study was data showing that males and females differed in the protein targets of, but not requirement for, linear polyubiquitination in the amygdala during fear memory formation. These data are similar to our recently reported result where we found that males and females differed in the regulation and engagement of, but not requirement for, protein degradation in the amygdala during fear memory formation (Devulapalli et al., 2021). While much still remains unknown about the role of sex in ubiquitin-proteasome signaling in the brain, our data strongly indicate that males and females may differ in the functional need for degradation-dependent and degradation-independent ubiquitin signaling in the amygdala during fear memory formation. Future studies will need to identify the protein targets of other forms of polyubiquitination (K48, K63) following fear conditioning and how this varies between males and females, which could provide critical insight into the role of sex in ubiquitin signaling during fear memory formation.

In conclusion, we found that sex-dependent, lysine-independent linear polyubiquitination was a critical regulator of fear memory formation in the amygdala. These data add to our rapidly expanding knowledge regarding the role of ubiquitin-proteasome signaling in memory formation and suggest that both proteasome-dependent and proteasome-independent protein polyubiquitination are critical regulators of learning-induced synaptic plasticity in the brain. These results also may have important implications for understanding the role of ubiquitin-proteasome dysregulation in neurological and neurodegenerative disorders.

## Data Availability Statement

The datasets presented in this study can be found in online repositories. The names of the repository/repositories and accession number(s) can be found below: https://www.ebi.ac.uk/pride/archive/, PXD025483.

## Ethics Statement

The animal study was reviewed and approved by Virginia Polytechnic Institute and State University Institutional Animal Care and Use Committee.

## Author Contributions

MM and TJ designed the experiments and wrote the manuscript. MM, KF, and SN conducted the experiments. WR and RH performed mass spectrometry analysis. All authors contributed to the article and approved the submitted version.

## Conflict of Interest

The authors declare that the research was conducted in the absence of any commercial or financial relationships that could be construed as a potential conflict of interest.
